# Evacuation solutions for individuals with functional limitations in the built environment: a scoping review protocol

**DOI:** 10.1186/s13643-021-01844-w

**Published:** 2021-12-20

**Authors:** Brad W. R. Roberts, Abdulrahman Al Bochi, Mark Weiler, Yashoda Sharma, Cesar Marquez-Chin, Steven Pong, Jessica Babineau, Waqas Sajid, Tilak Dutta, Albert H. Vette

**Affiliations:** 1 Department of Mechanical Engineering, University of Alberta, Donadeo Innovation Centre for Engineering, 9211 116 Street NW, Edmonton, Alberta T6G 1H9 Canada; 2 KITE – Toronto Rehabilitation Institute, University Health Network, 550 University Avenue, Toronto, Ontario M5G 2A2 Canada; 3 Department of Chemistry and Biology, Ryerson University, 350 Victoria Street, Toronto, Ontario M5B 2K3 Canada; 4 Wilfrid Laurier University, 75 University Avenue W, Waterloo, Ontario N2L 3C5 Canada; 5 Institute of Biomedical Engineering, University of Toronto, 164 College Street, Toronto, Ontario M5S 3G9 Canada; 6 Library & Information Services, University Health Network, 550 University Avenue, Toronto, Ontario M5G 2A2 Canada; 7 Glenrose Rehabilitation Hospital, Alberta Health Services, 10230 111 Avenue NW, Edmonton, Alberta T5G 0B7 Canada

**Keywords:** Buildings, Built environment, Canada, Egress solutions, Evacuation guidelines, Evacuation solutions, Functional limitations, Impairments, Scoping review, Scoping review protocol

## Abstract

**Background:**

Whether due to aging, disability, injury, or other circumstances, an increasing number of Canadians experience functional limitations that reduce their ability to participate in activities of daily life. While the built environment has become increasingly accessible, existing Canadian evacuation guidelines lack comprehensive strategies for evacuating individuals with functional limitations from buildings during emergencies. To inform guideline revisions, a map of existing solutions for evacuating such individuals is required. Therefore, this scoping review aims to provide an account of solutions that have been reported to safely evacuate individuals with functional limitations from the built environment.

**Methods:**

We will conduct a scoping review using the Arksey and O’Malley methodological framework. To identify potentially relevant studies, comprehensive searches (from January 2002 onwards) of the CINAHL, Ei Compendex, Inspec, Embase, MEDLINE, KCI, RSCI, SciELO CI, Web of Science Collection, and Scopus databases will be performed. Using a set of inclusion and exclusion criteria, two reviewers will independently (1) classify identified studies as relevant, irrelevant, or maybe relevant by evaluating their titles and abstracts and (2) classify the relevant and maybe relevant studies as included or excluded by evaluating their full-text. From each included study, data on publication information, study purpose, methodological details, evacuation information, and outcomes will be extracted using a set of data extraction items. We will present a numerical summary of the key characteristics of the included studies. For each evacuation activity, reported evacuation solutions will be summarized, and citations provided for functional limitations that are targeted by a given evacuation solution. To inform Canadian evacuation guideline revisions, we will tabulate evacuation activities common to different types of buildings and emergencies.

**Discussion:**

To our knowledge, this will be the first scoping review to identify the state and use of solutions for evacuating individuals with functional limitations from the built environment. Identifying solutions that enable all individuals to safely evacuate from different types of buildings will allow us to inform recommendations for the revision of evacuation guidelines in Canada and other jurisdictions. The findings of this scoping review will be published in a peer-reviewed journal, presented at relevant conferences, and made publicly available on the internet.

**Systematic review registration:**

Open Science Framework: osf.io/jefgy

**Supplementary Information:**

The online version contains supplementary material available at 10.1186/s13643-021-01844-w.

## Background

One in five Canadians aged 15 years and over experience impairments, and this proportion is expected to rise as our population ages [1]. Additionally, one in twenty children under the age of 15 years is impaired [2]. Types of impairments experienced are manifold and include those to cognitive, mental, physical, and sensory functions [1, 2]. A substantial number of individuals also find themselves in other needs-situations (e.g., pregnancy in women or critical illness in children) [3]. Such impairments and other needs that restrict the performance in fundamental physical and cognitive activities during daily life are generally termed *functional limitations* [4]. In this context, the built environment in Canada has become increasingly accessible over the past 10 to 15 years, and Canada has recently committed to the goal set out by the United Nations to provide universal access to public spaces by 2030 [5, 6]. A critical requirement for universal accessibility is the ability to safely evacuate all occupants from a space in the event of an emergency (e.g., fire or flood emergencies) [5]. Despite the importance of evacuation strategies for occupants with functional limitations, a review by the National Research Council of Canada found that little attention has been given to this topic [7]. In fact, the Canadian Commission on Building and Fire Codes recently recommended reviewing and revising requirements on evacuation strategies as a key target for future work [5].

The latest Canadian evacuation guidelines were published in 2002 by the National Research Council of Canada [8]. However, there has since been an increase in both the severity and chronicity of impairments that individuals live with outside of clinical settings [3]. Additionally, there have been advances in assistive technologies as well as an emergence of other technological innovations that can potentially facilitate the evacuation of individuals with functional limitations [9, 10]. Furthermore, it is important to note that existing Canadian guidelines focus exclusively on fire-related evacuations. As such, more comprehensive evacuation strategies are needed that also address other types of emergencies and for a range of functional limitations.

Based on the above considerations, there is an urgent need to revise existing Canadian guidelines for evacuating individuals with functional limitations from the built environment in emergencies. Such revised guidelines should further be tailored to the type of building, functional limitation, and emergency. In order to inform guideline revisions, it is important to first examine the current state and use of solutions for evacuating individuals with functional limitations. However, there has been, to our knowledge, no review of studies investigating this topic. The objective of this scoping review, therefore, is to capture and evaluate research on strategies and technologies for evacuating individuals with functional limitations from the built environment. The specific research question this scoping review will address is: What solutions have been reported that enable safe evacuation from the built environment for individuals with functional limitations?

## Methods

Our multidisciplinary review team has expertise in evacuation safety; the development of injury prevention, assistive, and rehabilitation technologies; and comprehensive literature searches, knowledge syntheses, and scoping reviews. For further information on the team, see the “Authors’ information” subsection of the “Declarations” section.

This scoping review protocol has been registered with the Open Science Framework (registration ID: osf.io/jefgy) and is being reported in accordance with the Preferred Reporting Items for Systematic Review and Meta-Analysis Protocols (PRISMA-P) statement [11]. The completed PRISMA-P checklist for this protocol can be found in Additional file 1. The performed scoping review will follow the Arksey and O’Malley six-stage methodological framework for scoping reviews [12]: (1) identifying the research question; (2) identifying relevant studies; (3) study selection; (4) charting the data; (5) collating, summarizing, and reporting the results; and (6) consulting with stakeholders. It will also be guided by studies that improved this framework [13, 14] and by the Joanna Briggs Institute Methodology for JBI Scoping Reviews [15]. The performed scoping review will be reported in accordance with the PRISMA extension for Scoping Reviews (PRISMA-ScR) statement [16].

### Identifying relevant studies

Based on our research question, this review will focus on studies reporting strategies and technologies for evacuating individuals with functional limitations from the built environment. To be included in this review, studies must report solutions for enabling individuals with functional limitations to evacuate from buildings in the event of an emergency. We will exclude studies if they report evacuation strategies only for individuals without functional limitations or if they report evacuation strategies for other elements of the built environment (e.g., parks or modes of transportation). The literature will be limited to original, peer-reviewed, journal articles and conference proceedings that have been written in English and published from the year 2002 onwards. Since the latest Canadian evacuation guidelines were published in 2002, literature published before this year will be excluded. However, we acknowledge that relevant evacuation solutions reported before 2002 and not considered in the latest Canadian evacuation guidelines may be missed.

To identify potentially relevant studies for inclusion, the following bibliographic databases will be comprehensively searched (from January 2002 onwards): CINAHL (via EBSCO); Ei Compendex and Inspec (via Engineering Village); Embase and MEDLINE (via Ovid); KCI, RSCI, SciELO CI, and the Web of Science Collection (via Web of Science); and Scopus (via Elsevier). The search strategy will be based on the inclusion criteria described above and will be developed by combining the main concepts of this scoping review: functional limitation, evacuation, and built environment. The search strategy is being developed in CINAHL and tailored for each additional database. It will include both keyword terms and controlled vocabularies (such as Medical Subject Headings (MeSH)), combined using Boolean operators. In cases where the equivalent of a controlled vocabulary term present in the CINAHL search string is not found in a subsequent database, the term will be removed for that particular database only. To ensure the search strategy optimizes the inclusion of relevant studies and the exclusion of irrelevant studies, it will also include Boolean NOT operators for keywords that are found in irrelevant studies only, but are related to the search concepts in some way (e.g., the term abortion for the evacuation concept). NOT terms will be unique to each database, given that different databases use different terminologies to describe the main concepts. The search strategy is being developed in consultation with an academic librarian (MW) and refined through team discussion. Since scoping reviews are iterative by nature [13], the search strategy will be refined until the most relevant search results are obtained. In refining the search strategy, we will hand-search the reference lists of relevant review studies and of articles in key journals (e.g., *Fire Technology*) to ensure relevant studies are not missed by the database searches. See Additional file 2 for a preliminary search strategy.

### Study selection

The final search results will be exported from the previously described databases and uploaded into Covidence (Covidence, Melbourne, VIC, AU), an online systematic review manager, where duplicate studies will be removed and the study selection as well as the data extraction processes will take place.

Using the inclusion criteria described above, a standardized set of exclusion criteria for study selection will be developed and tested on a random sample of fifty studies among the identified studies. A preliminary set of exclusion criteria is shown in Table 1. Four reviewers (AAB, BWRR, WS, and Zeyad Ghulam) will independently evaluate the titles and abstracts of the fifty studies using the exclusion criteria and classify each study as relevant, irrelevant, or maybe relevant. The studies classified as relevant or maybe relevant will then be independently evaluated at the full-text level by the four reviewers using, again, the exclusion criteria and classified as either included or excluded. For studies classified as excluded, the primary reason for study exclusion will be recorded. The exclusion criteria will be refined through discussion by the four reviewers until they agree on the classification of at least forty-five of the fifty studies. The four reviewers will then use the final exclusion criteria and the described study selection process to classify all identified studies. Each reviewer will classify half the identified studies (i.e., each study will be classified by two reviewers). At each stage of the screening (i.e., title and abstract as well as full-text screening), disagreements between reviewers will be resolved through adjudication by one of the other reviewers.

**Table 1:** Preliminary standardized study exclusion criteria

**Primary reason study is excluded**	
1. Full-text not available	
2. Not a journal article or conference proceeding	
3. Published before January 1, 2002	
4. Not written in English	
5. Not original research	
6. Not peer-reviewed	
7. Does not report evacuation solutions	
8. Reported evacuation solutions are for individuals without functional limitations	
9. Reported evacuation solutions are for non-building evacuations	

### Charting the data

A standardized set of data extraction items will be developed to extract key data from the included studies, allowing us to describe the studies and to answer our research question. Two authors (AAB and BWRR) will independently read, in depth, the full-text of each included study and extract relevant data. We anticipate that extracted data will include (1) publication information (e.g., title and first author), (2) study purpose (e.g., background and objectives), (3) methodological details (e.g., design and methodology), (4) evacuation information (e.g., evacuation solutions), and (5) outcomes (e.g., significant findings). A preliminary set of data extraction items is shown in Table 2. Throughout the data extraction process, the set of data extraction items will be refined and the extracted data updated as the team becomes familiar with the literature. Disagreements between the two authors will again be resolved through adjudication by a third team member (WS).

**Table 2:** Preliminary standardized data extraction items

**Publication information**	
Study title	
Type of study	
Source of study (e.g., journal name)	
First author	
Year of publication	
Country of study	
Type of funding	
**Study purpose**	
Background	
Objectives	
**Methodological details**	
Study design	
Summary of methodology	
**Evacuation information**	
Building types	
Emergency types	
Evacuation activities	
Functional limitations	
Evacuation solutions	
**Outcomes**	
Significant findings	
Conclusions	
Future research	

Consistent with both the Arksey and O’Malley framework [12] and the conduct of previous scoping reviews [17], no critical appraisal (i.e., systematic assessment of the validity) of the included studies will be performed.

### Collating, summarizing, and reporting results

The study selection process will be summarized narratively and using a flow diagram. We will present characteristics of the included studies in two ways. First, a numerical summary of the key characteristics of the studies will be presented in narrative and tabular form. The presented characteristics will include publication characteristics (e.g., study type, year of publication, geographic location, and funding type), methodological characteristics (e.g., study design), and evacuation solution characteristics (e.g., evacuation activities and functional limitations for which evacuation solutions are reported). This part of the analysis will reveal the dominant areas of research in terms of evacuation activities and functional limitations for which evacuation solutions are reported. Second, we will present a thematic summary of the studies in narrative and tabular form. Specifically, for each evacuation activity, reported evacuation solutions will be summarized, and citations provided for each relevant type of functional limitation (i.e., functional limitations that are targeted by a given evacuation solution). Functional limitations will be classified according to the *International Classification of Functioning, Disability and Health (ICF)* [18]. In order to inform recommendations for Canadian evacuation guideline revisions, we will also tabulate evacuation activities common to different types of buildings and emergencies. However, how to best report the results of a scoping review is an iterative process. Therefore, the described methodology for reporting the results will be refined throughout the review process. Finally, we expect to identify and report existing gaps in the literature (e.g., evacuation activities and corresponding functional limitations with no reported evacuation solutions) that require future research.

### Stakeholder consultation

To enhance this scoping review, we will rely on the expertise and experience of a broad range of key stakeholders. We will identify and invite individuals with functional limitations as well as their caregivers, emergency services personnel (e.g., firefighters and paramedics), and organizations that develop evacuation technologies to contribute to this scoping review by providing feedback on it. Potential stakeholders will be identified by AGE-WELL (Toronto, ON, CA) and the Ontario Neurotrauma Foundation (Toronto, ON, CA) using their extensive networks. Stakeholders will be invited to provide feedback on the scoping review during consultation meetings at several points throughout the review process.

## Discussion

To our knowledge, this will be the first scoping review to identify the state and use of solutions for evacuating individuals with functional limitations from the built environment. It will provide a comprehensive map of this topic and will inform the revision of Canadian guidelines for evacuating individuals with functional limitations from the built environment in emergencies. Commonly accepted scoping review processes will be followed, including the Arksey and O’Malley methodological framework and the PRISMA-ScR statement. By identifying evacuation solutions that enable all individuals to safely evacuate from different types of buildings, we anticipate that the results of this review will allow us to inform recommendations for the revision of evacuation guidelines in Canada and other jurisdictions. However, since we will not critically appraise the studies included in this scoping review, future work will be needed to assess their validity before recommendations for guideline revisions can be made. If important amendments are made to the protocol during the review process, they will be reported with the results of the scoping review. To effectively communicate our findings to policymakers and stakeholders, the results of this scoping review will be (1) published in a peer-reviewed journal, (2) presented at relevant national and international conferences, and (3) prepared in a report that will be written in both English and French in accessible formats (e.g., tagged Portable Document Format (PDF)) and shared through AGE-WELL, the Ontario Neurotrauma Foundation, the Toronto Rehabilitation Institute (Toronto, ON, CA), the University Health Network (Toronto, ON, CA), the Glenrose Rehabilitation Hospital (Edmonton, AB, CA), the University of Toronto Libraries TSpace Research Repository (Toronto, ON, CA), and the University of Alberta Education and Research Archive (Edmonton, AB, CA) by posting it to their respective websites.

## Supplementary Information

**Additional file 1:.** PRISMA-P checklist.

**Additional file 2:.** Preliminary search strategy.

## Data Availability

Data used and/or analyzed during this review will be available from the corresponding author upon request.

## Abbreviations

PRISMA-P: Preferred Reporting Items for Systematic Review and Meta-Analysis Protocols
PRISMA-ScR: PRISMA extension for Scoping Reviews
MeSH: Medical Subject Headings
ICF: International Classification of Functioning, Disability and Health
PDF: Portable Document Format
